# Robot-assisted laparoscopic resection of giant cystic pheochromocytoma: A case report

**DOI:** 10.1097/MD.0000000000045640

**Published:** 2025-11-07

**Authors:** Xiaolong Huang, Hang Tong, Junchi Chen, Youlin Kuang, Weiyang He

**Affiliations:** a Department of Urology, People’s Hospital of Chongqing Hechuan, Chongqing, China; b Department of Urology, The First Affiliated Hospital of Chongqing Medical University, Chongqing, China.

**Keywords:** adrenal gland, cystic, giant, pheochromocytoma, robot-assisted

## Abstract

**Rationale:**

The giant cystic pheochromocytomas (>10 cm) are exceedingly rare. Due to the massive size of tumor, traditional laparoscopic minimally invasive surgery poses significant technical challenges.

**Patient concerns:**

We present the case of a 53-year-old male patient who was admitted due to poorly controlled hypertension and an ultrasound-detected right adrenal mass.

**Diagnoses:**

Abdomen computed tomography scans revealed a cystic mass (11 cm × 9 cm) in the right adrenal gland, highly suggestive of pheochromocytoma. The laboratory findings demonstrate significantly elevated levels of catecholamines and their metabolites. Histopathological examination confirmed the diagnosis of cystic pheochromocytoma.

**Interventions:**

After 2 weeks of preoperative α-blockade preparation, the patient successfully underwent robot-assisted laparoscopic resection of the right adrenal tumor.

**Outcomes:**

At the 6-week postoperative evaluation, catecholamine levels had normalized. Blood pressure has returned to normal range in the absence of antihypertensive therapy and there are no more symptoms of dizziness and palpitations. The patient remained disease-free with no evidence of recurrence or metastasis during 1 year of surveillance.

**Lessons:**

In this case, we demonstrate the successful resection of a giant cystic pheochromocytoma in the right adrenal gland using robotic-assisted laparoscopy. This approach overcomes the technical limitations of conventional laparoscopy for large-volume adrenal pheochromocytomas and provides a novel therapeutic alternative for these challenging tumors.

## 1. Introduction

Pheochromocytoma is a rare neuroendocrine tumor that typically originates from the adrenal medulla, with an estimated incidence of 2 to 8 cases per million individuals.^[1]^ This tumor is characterized by excessive catecholamine secretion, resulting in a variety of clinical manifestations, including hypertension, headaches, palpitations, and diaphoresis, etc. Cystic pheochromocytoma is regarded as a unique subtype caused by myxoid degeneration or posthemorrhagic necrosis. And the cystic form of pheochromocytoma is even rarer, accounts for approximately 20% of all cases.^[2,3]^ In clinical practice, adrenal tumors with a size ranging from 6 to 10 cm are considered large, while those >10 cm are classified as giant tumors.^[4]^ Surgical resection remains the primary clinical treatment for adrenal pheochromocytoma. Laparoscopic adrenalectomy and robot-assisted laparoscopic adrenalectomy are the 2 main minimally invasive surgical methods. Given the adrenal glands’ retroperitoneal location and their deep anatomical position, the operative space is considerably constrained. Traditional laparoscopic surgery presents significant technical challenges due to limited instrument maneuverability and 2-dimensional visualization. Recent study has demonstrated that robotic-assisted laparoscopic resection offers distinct advantages for managing complex cases and large-volume adrenal tumors.^[5]^ Here, we present a case of robotic-assisted laparoscopic resection of a giant cystic pheochromocytoma in the Department of Urology, the First Affiliated Hospital of Chongqing Medical University. The robotic platform utilized was the da Vinci Xi Surgical System (Intuitive Surgical, Sunnyvale).

## 2. Case presentation

**Figure 1.**
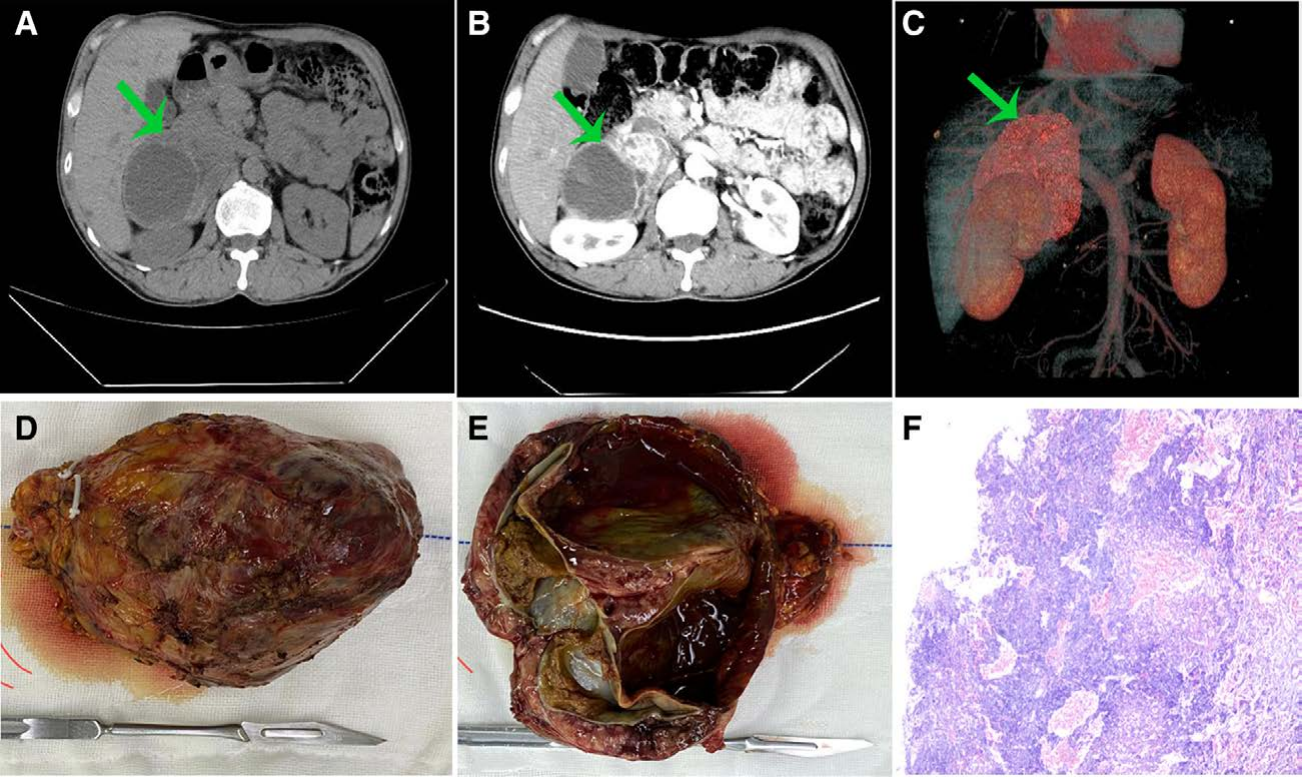
(A) Abdominal non-contrast CT scan: a huge mass was shown in the right adrenal area, size 10 × 9 cm. (B) Abdominal contrast-enhanced CT scan: the mass shows significant enhancement after contrast administration, containing a round hypodense focus. (C) Three-dimensional stereoscopic image of the right adrenal mass. (D) A mass that has been completely excised,the size was measured to be about 11 × 10 × 9 cm. (E) The mass contains internal cystic components. (F) Tumor histopathology (HE ×100): Pheochromocytoma with cystic changes. CT = computed tomography.

The patient was a 53-year-old male with a history of hypertension for 5 years. Due to poor blood pressure control, color ultrasound was performed in another hospital and found that the right adrenal mass was hospitalized in our department. During the course of the disease, the maximum blood pressure of the patient was 210/110 mm Hg, with significant blood pressure fluctuations. These hypertensive episodes were typically accompanied by palpitations and dizziness, but notably without severe headache or diaphoresis. The results of detection of catecholamines and their metabolites after admission are shown in Table 1. Abdomen computed tomography revealed a right adrenal mass measuring approximately 11 cm × 9 cm, with a roundish hypodense lesion within it. Radiologic features raised the possibility of pheochromocytoma (Fig. 1A–C). This patient demonstrated a strong preference for minimally invasive surgery. For preoperative optimization given the presumed pheochromocytoma, the patient was started on oral phenoxybenzamine for 2 weeks and blood volume expansion for 3 days. When all the indicators of the patient met the surgical standards, robot-assisted laparoscopic resection of the right adrenal giant mass was performed.

**Table 1:** Preoperative catecholamines and their metabolites.

	Actual value (pg/mL)	Normal range (pg/mL)
Norepinephrine	3185.70	217–1109
Epinephrine	813.50	<95
Dopamine	42.40	<20
Metanephrine	2274.5	<62
Normetanephrine	2332.3	<145
3-Methoxytyramine	16	<18.4

General anesthesia was induced with endotracheal intubation. Intraoperative monitoring included real-time invasive arterial blood pressure and central venous pressure, and a multi-channel intravenous access was established for fluid administration. Due to the enormous tumor size, the transperitoneal approach was adopted. The patient was in a left oblique supine position. The robotic surgical operation platform and special instruments were placed in appropriate positions, and the surgical cavity was dilated to 14 mm Hg with CO_2_. A huge tumor was seen in the right adrenal area. Its upper boundary was closely attached to the liver. The tumor was located behind the inferior vena cava and had adhesions, compressing the vena cava and the right renal vein. The surface of the tumor was rich in nourishing blood vessels. Gradually free and separate the adhered sites along the tumor surface. During the separation process, there was obvious bleeding. Hem-o-lok clamped the tumor-nourishing vessels and adrenal vessels in time to stop the bleeding. After the tumor was completely free, the tumor of the right adrenal gland and part of the adrenal tissue were resected, while some normal adrenal tissues were retained. The intraoperative blood pressure fluctuation range was 140–230/60–120 mm Hg, and the bleeding was approximately 400 mL. The operation time was 285 minutes. The tumor size was 11 cm × 10 cm × 9 cm, and cystic lesions were observed (Fig. 1D, E). Postoperative pathological diagnosis showed pheochromocytoma with cystic changes (Fig. 1F). Immunohistochemistry: CK(−), CgA(+), Syn(+), CD56(+), SF-1(−). The levels of catecholamines and their metabolites were reexamined 6 weeks after the operation and all returned to normal. The results are shown in Table 2. The blood pressure has normalized without antihypertensive medication and there are no more symptoms of dizziness and palpitations. The patient has been followed up for 1 year after the operation, and no recurrence or metastasis occurred.

**Table 2:** Results of catecholamines and their metabolites 6 weeks after the operation.

	Actual value (pg/mL)	Normal range (pg/mL)
Norepinephrine	376.7	217–1109
Epinephrine	93.2	<95
Dopamine	3.7	<20
Metanephrine	45.3	<62
Normetanephrine	84.8	<145
3-Methoxytyramine	8	<18.4

## 3. Discussion

Giant adrenal pheochromocytoma is rare, and the cystic form is even rarer. Surgical resection is currently the most effective method for the treatment of pheochromocytoma. Because catecholamines may be released during tumor surgery, causing drastic hemodynamic changes, especially tumors of giant volume, this surgery is also recognized as a high-risk operation. Adequate preoperative preparation, anesthesia management and intraoperative reduction of tumor compression are important factors to ensure surgical safety.^[6,7]^ The diagnosis of pheochromocytoma relies on the determination of catecholamine levels as well as imaging computed tomography and magnetic resonance imaging. However, studies have demonstrated that cystic pheochromocytomas may contain high concentrations of catecholamines within their cystic fluid.^[8,9]^ Due to the encapsulating cystic architecture, these hormones often remain sequestered rather than being released into systemic circulation, potentially yielding falsely normal plasma catecholamine metabolite levels. Without adequate preoperative preparation, surgical manipulation of cystic pheochromocytoma may cause massive release of catecholamines from the cyst into circulation, potentially triggering severe hemodynamic instability and life-threatening arrhythmias. Therefore, comprehensive preoperative optimization is strongly recommended for suspected pheochromocytoma cases, including blood pressure control, heart rate management, and blood volume expansion, etc.^[10]^ Due to the enormous tumor size of the pheochromocytoma in this case, the patient underwent 2 weeks of preoperative preparation to meet the criteria for surgery. Although intraoperative blood pressure fluctuations occurred, which were effectively managed and stabilized by the anesthesiologist using urapidil.

Since laparoscopic adrenalectomy was first reported in 1992, laparoscopic surgery has been widely used for adrenal tumors.^[11]^ However, large adrenal tumors often present with significant peritumoral adhesions, making dissection technically challenging. The limited operative space and prolonged surgical duration further increase the complexity of laparoscopic resection.^[12]^ Due to the high-risk nature of pheochromocytoma surgery, open resection has historically been the recommended approach for large tumors (>6 cm).^[13]^ Nevertheless, open surgery is a more extensive procedure that involves a significant incision, typically measuring more than 20 cm, which leads to a longer recovery time. Moreover, Kiernan et al^[7]^ demonstrated that the open surgery significantly increased intra- and postoperative hemodynamic instability. Recent evidence has suggested that laparoscopic surgery could also be considered for large-volume adrenal pheochromocytoma, particularly when performed by surgeons with extensive laparoscopic experience. This approach can achieve outcomes comparable to those of open surgery while reducing the incidence of perioperative complications.^[14,15]^ With the development of minimally invasive concepts and equipment technologies, robot-assisted laparoscopic adrenalectomy has been increasingly adopted in clinical practice in recent years. Robot assisted surgery offers distinct advantages with its flexible robotic arms and high-definition 3D magnified visualization.^[16]^ This technology excels in operating within anatomical blind spots and confined spaces that challenge conventional laparoscopy, while minimizing tissue manipulation. Furthermore, the robotic system’s tremor filtration capability significantly reduces intraoperative vascular injuries by automatically compensating for hand tremors.^[17]^ Additionally, research suggested that robot-assisted laparoscopy for large adrenal tumors (>6 cm) may be superior to traditional laparoscopy, with benefits such as less intraoperative bleeding, fewer conversions to open surgery, and quicker recovery of gastrointestinal function.^[18,19]^ In our view, extensive experience in laparoscopic or robotic surgery is crucial for effectively reducing surgical complications. A multicenter study reported by Azhar et al^[20]^ demonstrated that minimally invasive approaches (including laparoscopic and robotic techniques) for large adrenal tumors (>5 cm) can achieve outcomes comparable to those of traditional open surgery, with fewer complications. The study further emphasized that robotic surgery offers particular advantages in the resection of giant adrenal tumor. Our surgeons are well-versed in performing da Vinci robotic-assisted procedures. After a thorough discussion of the associated risks and alternative treatment options, the patient voluntarily chose to undergo robotic-assisted laparoscopic surgery.

Adrenal tumor resection is typically performed via either the transperitoneal or posterior retroperitoneal approach. A meta-analysis demonstrated that the posterior retroperitoneal approach is more suitable for resection of smaller adrenal tumors.^[21]^ However, for obese patients or large pheochromocytomas (>6 cm), the transperitoneal approach is preferred due to the limited working space in retroperitoneal access, which may compress the tumor and potentially induce catecholamine release, precipitating intraoperative crisis.^[22,23]^ Although the size of the tumor is an important reference factor for choosing a surgical approach, surgeons can also make a comprehensive assessment based on the adjacent relationship around the tumor and their own experience and habits.

Moreover, the appropriate extent of normal adrenal tissue preservation remains a contentious issue in surgical practice. Although pheochromocytoma can exhibit malignant behavior characterized by metastasis, only a minority of patients (15–17%) ultimately develop metastatic disease.^[24]^ Approximately 37% of patients undergoing unilateral total adrenalectomy develop hypocortisolism postoperatively, with some even experiencing iatrogenic adrenal insufficiency requiring lifelong steroid replacement therapy.^[25]^ In recent years, adrenal-sparing surgery for pheochromocytoma has gained increasing preference. However, residual adrenal tissue still carries risks of tumor recurrence or metastasis, and may necessitate more complicated reoperations.^[26]^ Simone et al^[27]^ reported that tumor location served as a crucial determinant in deciding whether to preserve normal adrenal tissue. For patients undergoing adrenal-sparing surgery, postoperative metanephrine levels should be reevaluated 2 to 6 weeks after the procedure to assess surgical completeness. If elevated metanephrine levels are detected at any follow-up point, radiographic evaluation is recommended within 3 months postoperatively.^[13]^ In this case, based on preoperative imaging findings and the patient’s preference, we opted for a pheochromocytoma resection with preservation of partial normal adrenal tissue. Postoperative follow-up results have demonstrated satisfactory outcomes.

## 4. Conclusion

Giant adrenal cystic pheochromocytoma represents an exceptionally rare occurrence. In this case report, we achieved complete resection of a giant cystic pheochromocytoma from the right adrenal gland using robot-assisted laparoscopy through a transperitoneal approach, confirming the feasibility of robotic surgery for giant adrenal cystic pheochromocytomas. Given the rarity of giant adrenal cystic pheochromocytomas, current evidence remains limited to case reports, with scarce data available for comprehensive analysis. Therefore, large-scale multicenter studies are still needed to further confirm that robot-assisted surgery has advantages over traditional laparoscopic surgery for giant adrenal cystic pheochromocytoma.

## Acknowledgments

The authors thank the patient for granting consent to publish this case report.

## Author contributions

**Conceptualization:** Xiaolong Huang, Weiyang He.

**Data curation:** Hang Tong.

**Funding acquisition:** Weiyang He.

**Supervision:** Youlin Kuang.

**Writing – original draft:** Xiaolong Huang, Junchi Chen.

**Writing – review & editing:** Youlin Kuang.
